# Influence of Social Support on Peri‐Operative Pain: A Secondary Sub‐Sample Analysis of a Prospective Observational Trial

**DOI:** 10.1002/ejp.70094

**Published:** 2025-07-28

**Authors:** Philipp Wenzel, Marthe Gründahl, Monika Fischer, Grit Hein, Fabian Fuchtmann, Kathrin Schnabel, Daniela C. Rosenberger, Esther M. Pogatzki‐Zahn, Heike L. Rittner, Karolin Teichmüller

**Affiliations:** ^1^ Department of Anaesthesiology, Intensive Care, Emergency and Pain Medicine, Centre for Interdisciplinary Pain Medicine Würzburg University Hospital Würzburg Würzburg Germany; ^2^ Department of Psychiatry University Hospital Würzburg Würzburg Germany; ^3^ Department of Anaesthesiology, Intensive Care and Pain Medicine University Hospital Münster Münster Germany; ^4^ Department of Psychosomatic Medicine and Psychotherapy University Hospital Münster Münster Germany; ^5^ Department of Psychology University of Würzburg Würzburg Germany

**Keywords:** acute postsurgical pain, biopsychosocial model of pain, chronic postsurgical pain, Multidimensional Scale of Perceived Social Support (MSPSS), post‐operative pain, social buffering

## Abstract

**Background:**

Research on the role of social support in the context of acute pain is lacking, although a possible correlation between social support and acute pain might imply consequences for clinical practice. We expected perceived social support to predict the intensity of acute postsurgical pain and the dose of opioids given.

**Methods:**

Within a multicentre prospective observational study, *n* = 217 adult patients undergoing major surgery were recruited at the University Hospital of Würzburg with an additional questionnaire for assessing perceived social support using the Multidimensional Scale of Perceived Social Support (MSPSS). We also measured pre‐operative pain, opioid intake and psychological factors, such as depressiveness or pain catastrophising. Main outcomes were pain intensities at rest and at movement, and morphine equivalent on Day 1 and Day 7 after surgery.

**Results:**

Perceived social support was higher in general than expected from previous studies and depended on marital status. Certain socio‐demographic characteristics, pre‐operative chronic pain and depressive symptoms also correlated with perceived social support. Using multiple linear regression, we could confirm known risk factors for high intensity acute postsurgical pain, for example, younger age and pre‐operative pain. Perceived social support, however, was not a significant predictor of neither postsurgical pain intensity nor postsurgical opioid requirements.

**Conclusions:**

Perceived social support measured by MSPSS does not add to the prediction of acute postsurgical pain in our study. Acute pain might be influenced more strongly by biological and psychological factors than social factors. More fine‐grained measures might be necessary to record, for example, daily social support.

**Significance:**

Although social interactions are known to influence pain perception, studies on the role of social support on post‐operative acute pain are lacking. In a broad spectrum of surgeries, we found that perceived social support was positively related to pre‐operative health measures, for example, lower average pain intensity over the past 3 months or lower morphine equivalent and opioid intake before surgery. Perceived social support, however, was not a significant predictor of neither postsurgical pain intensity nor postsurgical opioid requirements.

## 1 Introduction

In about 18 M surgeries per year in Germany (Geil et al. 2019), 85% of patients experience acute pain. At least 12% of patients classify it as ‘severe to extreme’ at discharge (Buvanendran et al. 2015; Gan et al. 2014). Severe intensity and long duration of pain lead to lower patient satisfaction, more complications, a higher mortality, and, finally, chronic postsurgical pain (Bair et al. 2003; Schnabel et al. 2020; van Driel et al. 2022). Existing risk scores for severe acute post‐operative pain include young age, female sex, pre‐operative opioid use and pre‐operative pain, smoking, history of depression and sleep disturbances (Schnabel et al. 2020; Yang et al. 2019). However, the role of social support is unclear.

In chronic pain, psychosocial factors play a key role in its development: Factors with particularly good evidence include depression, anxiety disorders and pain catastrophising (Pogatzki‐Zahn 2021). Regarding social factors, the biosocial model suggests that emotionally supportive behaviour or validation might positively affect pain adjustment (i.e., decreased pain intensity and behaviour) by reducing negative emotional arousal (Linehan 1993). While not all studies provide significant results, more social support and social buffering are associated with reduced pain intensity, decreased physical disability and better psychological functioning, supporting the biosocial model and suggesting social support as a resilience factor (Andrejeva et al. 2021; Jensen et al. 2011; Weiss, Gründahl, et al. 2024). However, overly solicitous behaviour by the social environment is associated with the opposite outcome (Jensen et al. 2011). Similar to chronic pain, social support might be a protective factor in acute pain: In experimental settings, feeling socially isolated enhanced pain intensity, while social support, for example holding the partner's hand or looking at a picture of the partner, diminished pain intensity (Eisenberger 2012; Master et al. 2009). In patients undergoing breast cancer surgery, low social support predicted acute pain unpleasantness but showed no effect on pain intensity (Munk et al. 2023). Most studies assess perceived social support on a general level, using questionnaires such as the Multidimensional Scale of Perceived Social Support (MSPSS) scale. Here, patients answer questions regarding their social environment, for example whether they receive social support from their friends and family (Zimet et al. 1988).

Post‐operative pain is particularly interesting when examining the connection of perceived social support (PSS) and acute pain in more detail: Post‐operative pain can be scheduled, but it occurs in a more naturalistic setting than experimentally induced pain. Observations on post‐operative pain might thus have higher external validity. Moreover, a possibly positive effect of PSS on post‐operative pain could have important implications for pain treatment and prevention since patients with unfavourable psychosocial characteristics might benefit from a targeted intervention before and after surgery.

This study aims to shed light on the influence of PSS assessed by the MSPSS on peri‐operative pain. First, we wanted to investigate if PSS in patients undergoing surgery is associated with pre‐operative pain, pre‐operative pain medication and psychological factors. Second, we asked whether PSS is associated with more beneficial post‐operative outcomes and whether it contributes to the prediction of post‐operative acute pain intensity beyond known risk factors (Schnabel et al. 2020; Yang et al. 2019). We hypothesised that higher levels of social support (1) benefit pre‐operative functioning (i.e., less pain, less pain medication and better psychological status) and (2) show protective effects on acute pain following trauma and reduce post‐operative opioid use.

## 2 Methods

### 2.1 Study Design

The prospective cohort study was performed in the University Hospital of Würzburg, Germany, as part of a multicentre study. Among the six University Hospitals involved in the multicentre study, only the Würzburg site assessed perceived social support in addition to the core measures of the multicentre trial. The multicentre study was registered in the German Clinical Trials Register (DRKS). However, the present analysis, which constitutes a secondary evaluation of a sub‐sample from the larger study, was not prospectively pre‐registered. Ethical approval (154/21_z) was obtained from the Ethics Committee of the University of Würzburg on 26 July 2021. The study is reported according to the STROBE statement (Vandenbroucke et al. 2007).

### 2.2 Participants

Patient enrolment took place between November 2021 and April 2022. Patient inclusion and exclusion were based on the study protocol for the multicentre trial. Briefly, we included patients who met the criteria of age of majority (≥ 18 years), capacity to consent and good understanding of the German language. We approached mainly, but not exclusively, patients with major thoracic or abdominal surgery. Patients were mainly approached before or after their pre‐operative appointment with the anaesthesiologist and partly on the ward. All patients gave written informed consent to participate in the study.

### 2.3 Measurements

Data in the acute peri‐operative setting were obtained at three time points: before surgery at study inclusion, on the first post‐operative day (POD1) and on the seventh post‐operative day (POD7). Presurgical questionnaires were completed on paper immediately following inclusion, whereas POD1 and POD7 surveys were conducted as interviews, either in person or by telephone, depending on whether the patient had already been discharged.

### 2.4 Pre‐Operative Information

For assessing PSS, we used the MSPSS (Zimet et al. 1988), which was administered once before surgery together with a battery of other diagnostic tools. The MSPSS is short and easy to use, with its 12 items assessing social support from the family (e.g., ‘I can talk about my problems with my family’), friends (e.g., ‘My friends really try to help me’) and significant others (‘There is a special person who is around when I am in need’) with four items each. Items are scored on a 7‐point Likert scale from 1 (‘strongly disagree’) to 7 (‘strongly agree’). The total score is the average of the 12 items, with higher values indicating higher PSS. In addition to the total score, family, friends and significant others can be distinguished as specific sources of social support (Bruwer et al. 2008; Santiago et al. 2021; Zimet et al. 1988). Good reliability and validity have been confirmed in several studies (Bruwer et al. 2008; Dahlem et al. 1991; Santiago et al. 2021). In the current study, the scale showed excellent internal consistency with Cronbach's alpha of > 0.9 for the total score and all subscales.

All other questionnaires were used according to the multicentre study protocol. Depression and pain catastrophising were assessed using the 9‐item Patient Health Questionnaire (PHQ‐9; Spitzer et al. 1999) and the Pain Catastrophizing Scale (PCS; Sullivan et al. 1995), respectively. Patients rated their fear of surgery and anaesthesia on a numeric rating scale (NRS) ranging from 0 (‘no fear’) to 10 (‘greatest fear imaginable’). Sleep quality of the last 4 weeks before study inclusion was evaluated by answering a dichotomous question (‘Overall, have you slept poorly in the last four weeks?’). Pain‐related impairment was assessed using the Chronic Pain Grade Scale (CPGS; Von Korff et al. 1992). As part of the Luebeck Pain Risk Questionnaire (Vahldieck et al. 2018), patients were asked to rate their pain intensity over the last 3 months, as well as their expected pain intensity after surgery on an NRS from 0 (‘no pain’) to 10 (‘most intense pain imaginable’). Pain medication prior to surgery, especially opioid use, was enquired. The conversion to morphine equivalent dose was performed using the app ‘Opioid Calculator’ (ANZCA 2020). Socio‐demographic characteristics included sex, age and marital status.

### 2.5 Post‐Operative Information

The duration of the surgery was measured from incision to suture and taken from the surgical protocol. The QUIPS questionnaire (Quality Improvement in Post‐operative Pain Management), which had proven successful in a quality management project performed in German hospitals, was used to assess pain on POD1 and POD7 (Meissner et al. 2008). Patients reported their pain intensities at rest and at movement on a NRS ranging from 0 (‘no pain’) to 10 (‘most intense pain imaginable’). Pain medication was taken from the clinic system or enquired on POD1 and POD7, and opioids were converted to morphine equivalent dose.

### 2.6 Data Analysis

**FIGURE 1:**
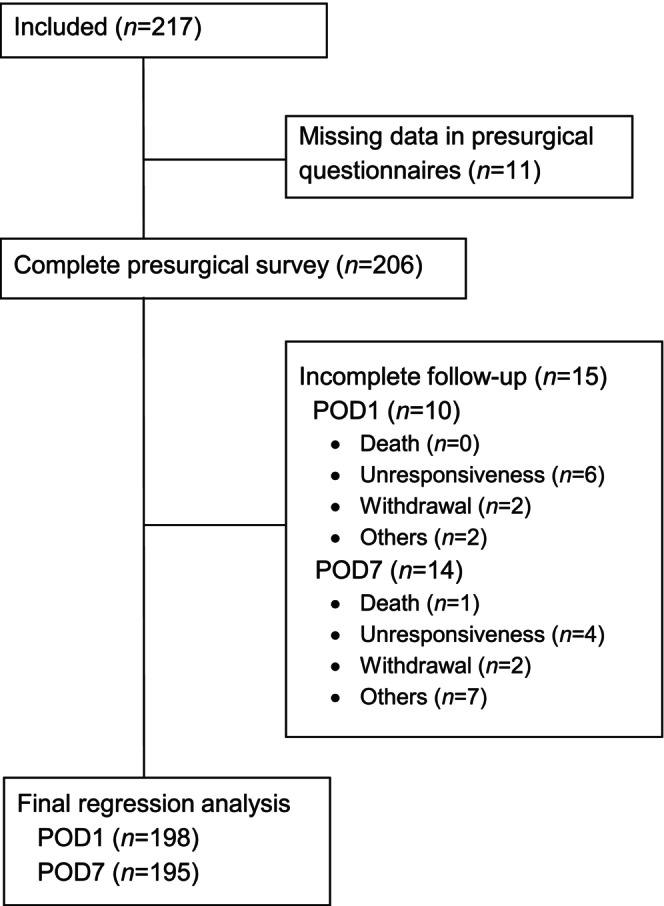
Flow chart of the analysis.

All data were analysed using the SPSS software package (IBM SPSS Statistics Version 27.0). Tables and figures were created with Microsoft Word and Microsoft Excel (Version 16.0). The significance level was set to *α* = 0.05. Data are presented as mean ± SD, and *n* (%). Of all included patients, those with missing data were excluded from the analysis in question, whereas other fully completed questionnaires from the same patients were still used (Figure 1).

To test our hypothesis about a possible association of PSS and pre‐operative parameters, we calculated Spearman's correlation coefficients and Eta coefficients depending on the variables scaling. *p* values were adjusted according to Benjamini and Hochberg (1995) to control for multiple testing. To test our hypothesis about PSS as a possible predictor of post‐operative measures, we performed a multiple regression analysis for each of the following outcome variables: pain intensity at rest, pain intensity at movement and morphine equivalent on POD1 and POD7. Predictive variables were MSPSS score and those that have already been proposed as risk factors (Schnabel et al. 2020; Yang et al. 2019), specifically sex, age, duration of surgery, fear of surgery and anaesthesia, expected pain intensity, PHQ‐9 score, PCS score, sleep quality of the last 4 weeks, average pain intensity over the last 3 months and morphine equivalent taken prior to surgery. All models were checked for autocorrelation, multicollinearity, outliers and homoscedasticity. Bootstrapping was used when homoscedasticity was found to be insufficient, and outliers were excluded from the analysis if they were identified as such in more than one procedure. For four of the six regression models, bootstrapping with 2000 samples and a 95% confidence interval was applied to account for heteroskedasticity.

## 3 Results

### 3.1 Patient Cohort

A total of *n* = 217 patients were included, with only a few having missing data (approximately 10%, Figure 1).

An overview of socio‐demographic characteristics, as well as the types of surgery, is reported in Table 1. The different types of surgery resulted in a heterogeneous sample overall, but patients who declined to participate showed a similar distribution of sex and age to those included.

**TABLE 1:** Socio‐demographic data, surgeries, pain level and opioid dose.

Total sample, *n* (%)	217 (100%)
Sex, *n* (%)	
Male	120 (55.3%)
Female	97 (44.7%)
Age (years), *n* (%)	
18–30	11 (5.1%)
31–50	48 (22.1%)
51–70	119 (54.8%)
> 70	39 (18.0%)
Total (*M* ± SD)	57.6 ± 14.4
Marital status, *n* (%)	
Single	24 (11.1%)
Divorced	10 (4.6%)
Firm relationship	173 (79.7%)
Widowed	10 (4.6%)
Types of surgery, *n* (%)	
Gastrointestinal	69 (31.8%)
Urological	58 (26.7%)
Thoracic	37 (17.1%)
Thyroid gland	19 (8.8%)
Hernia repair	13 (6.0%)
Others	21 (9.7%)
Pain and opioids	
Pre‐op (*M* ± SD)	
Pain last 3 months	2.6 ± 2.9
Morphine equivalent (mg)	3.2 ± 16.2
POD1 (*M* ± SD)	
Pain at rest	2.3 ± 2.2
Pain at movement	4.8 ± 2.4
Morphine equivalent (mg)	27.0 ± 36.1
POD7 (*M* ± SD)	
Pain at rest	1.1 ± 1.5
Pain at movement	3.3 ± 2.3
Morphine equivalent (mg)	10.3 ± 24.6

Abbreviation: POD, post‐operative day.

### 3.2 High Level of PSS Correlates With Socio‐Demographic and Psychosocial Factors

Most patients reported high levels of social support, with an average MSPSS score of 6.20 ± 1.02. Thirty‐six patients (16.6%) suffered from moderate to severe symptoms of depression with a PHQ‐9 score > 9 (Kroenke et al. 2001), and 24 patients (11.2%) reported clinically relevant pain catastrophising with a PCS score > 29 (Sullivan 2009).

Table 2 shows mean scores of MSPSS, PHQ‐9, PCS and CPGS for the total sample and depending on socio‐demographic characteristics. It stands out that the MSPSS score of separated or divorced patients was significantly lower compared with all other relationship statuses (*t*_(214)_ = −4.83, *p* < 0.001), whereas patients in a firm partnership or marriage had the highest MSPSS scores. Scores representing depression, pain catastrophising and pain‐related impairment were lower in firmly related patients.

**TABLE 2:** Overview of several scores and their mean values depending on demographic characteristics.

Score (range)	All patients	Sex		Age (years)				Marital status			
		m	w	18–30	31–50	51–70	> 70	Single	Divorced	Firm relationship	Widowed
MSPSS (1–7)	6.2 ± 1.02	6.25 ± 0.90	6.13 ± 1.15	6.55 ± 0.85	6.21 ± 1.19	6.17 ± 0.99	6.16 ± 0.93	5.84 ± 1.04	4.75 ± 1.38	6.35 ± 0.89	5.89 ± 1.33
PHQ‐9 (0–27)	5.44 ± 4.87	4.71 ± 4.75	6.35 ± 4.88	6.18 ± 5.00	6.33 ± 5.83	5.39 ± 4.62	4.31 ± 4.14	7.58 ± 6.03	9.10 ± 8.03	4.97 ± 4.30	4.90 ± 5.07
PCS (0–52)	13.35 ± 10.51	12.15 ± 10.64	14.82 ± 10.20	17.09 ± 13.66	13.29 ± 10.25	13.37 ± 10.35	12.29 ± 10.52	15.17 ± 12.32	16.00 ± 12.17	12.79 ± 10.18	15.80 ± 10.05
CPGS (0–4)	1.24 ± 1.29	1.05 ± 1.20	1.50 ± 1.36	1.70 ± 1.34	1.41 ± 1.32	1.06 ± 1.19	1.44 ± 1.48	1.55 ± 1.36	1.90 ± 1.60	1.11 ± 1.21	2.10 ± 1.52

*Note:* Data are mean ± SD.

Abbreviations: CPGS, Chronic Pain Grade Scale; MSPSS, Multidimensional Scale of Perceived Social Support; PCS, Pain Catastrophizing Scale; PHQ‐9, Patient Health Questionnaire.

The MSPSS score was associated with pre‐operative chronic pain, its treatment with opioids and symptoms of depression and pain catastrophising (Table 3): As expected, higher PSS correlated weakly, but significantly with lower average pain intensity in the last 3 months, lower morphine equivalent and opioid intake before surgery, better quality of sleep, as well as lower PHQ‐9, PCS and CPGS scores.

**TABLE 3:** Correlations between MSPSS mean values and other variables.

MSPSS × variable	*r*_s_	*η* ^2^
Age	−0.114	
Duration of surgery	−0.031	
Average pain intensity last 3 months	−0.222**	
Fear of surgery	−0.029	
Expected pain intensity	−0.044	
Morphine equivalent before surgery	−0.186*	
PHQ‐9 score	−0.266***	
PCS score	−0.165*	
Opioid intake before surgery	−0.183*	
CPGS	−0.197*	
Sex		0.003
Marital status		0.130***
Sleep		0.045**
Pain at rest POD1	−0.072	
Pain at rest POD7	−0.200*	
Pain at movement POD1	−0.077	
Pain at movement POD7	−0.068	
Morphine equivalent POD1	−0.176*	
Morphine equivalent POD7	−0.015	

*Note:* To measure correlation, we used Spearman and Eta coefficient for at least ordinal and nominal data, respectively. *p* values adjusted according to Benjamini and Hochberg (1995). Significance was tested two‐tailed.

Abbreviations: CPGS, Chronic Pain Grade Scale, MSPSS, Multidimensional Scale of Perceived Social Support; PCS, Pain Catastrophizing Scale; PHQ‐9, Patient Health Questionnaire; POD, post‐operative day.

* *p* < 0.05.

** *p* < 0.01.

*** *p* < 0.001.

### 3.3 Predictors of Post‐Operative Pain

Mean acute postsurgical pain levels and morphine equivalent doses are reported in Table 1. Across all surgeries in this sub‐cohort included in Würzburg, 49 (23.7%) patients reported relevant pain at rest of > 3, and 72 (34.8%) reported relevant pain at movement of > 5 on POD1. On POD7, 16 (7.9%) patients reported relevant rest pain of > 3, and 35 (17.2%) reported relevant pain at movement of > 5.

Significant zero‐order correlations emerged for the MSPSS with two of the postsurgical outcome measures (Table 3), indicating a weak association of higher PSS and lower pain at rest on POD7 and less opioid intake on POD1, respectively.

The predictive value of PSS and other risk factors was tested with six regression models, all of which produced significant results with varying goodness‐of‐fit. In the prediction of post‐operative pain and post‐operative morphine equivalent, we could confirm known risk factors in different compositions (Table 4): The most important predictor of high intensity acute pain was chronic pre‐operative pain, followed by long duration of surgery, young age and fear of surgery and anaesthesia. High post‐operative morphine equivalent doses were mainly predicted by pre‐operative opioid intake and high expected pain intensities, as well as poor sleep quality. PSS did not significantly predict any of the post‐operative pain outcome measures.

**TABLE 4:** Results of multiple linear regression.

Predictor	Pain at rest^a^				Pain at movement				Morphine equivalent^a^			
	POD1^b^		POD7^c^		POD1^d^		POD7^e^		POD1^f^		POD7^g^	
	*B*	*β*	*B*	*β*	*B*	*β*	*B*	*β*	*B*	*β*	*B*	*β*
MSPSS score	−0.146	−0.068	−0.186	−0.129	−0.121	−0.054	0.034	0.016	−2.995	−0.085	1.399	0.059
Sex	0.262	0.059	0.302	0.100	0.150	0.032	0.439	0.097	−3.405	−0.047	5.989	0.120
Age (years)	−0.029	−0.185*	−0.002	−0.021	−0.034	−0.212**	0.003	0.022	0.191	0.076	0.127	0.073
Duration of surgery (min)	0.006	0.225*	0.003	0.146	0.009	0.320***	0.004	0.148	0.051	0.119	0.034	0.117
Fear of surgery and anaesthesia	0.023	0.028	0.001	0.002	0.163	0.187*	0.032	0.038	−1.640	−0.122	−0.422	−0.045
Expected pain intensity	0.051	0.051	0.038	0.056	0.025	0.023	0.049	0.048	4.930	0.301**	1.641	0.146*
PHQ‐9 score	0.073	0.160	0.016	0.053	0.025	0.051	0.033	0.072	−0.046	−0.006	−2.496	−0.049
PCS score	−0.023	−0.111	0.004	0.026	−0.019	−0.088	0.016	0.074	0.075	0.022	0.280	0.055
Sleep quality last 4 weeks	−0.259	−0.057	0.156	0.051	−0.296	−0.061	−0.241	−0.052	−11.552	−0.156*	0.084	0.036
Avg. pain intensity last 3 months	0.254	0.326**	0.126	0.239**	0.233	0.284***	0.167	0.210*	1.173	0.092	0.228	0.026
Morphine equivalent prior to surgery (mg)	−0.009	−0.068	0.005	0.058	0.005	0.037	0.006	0.047	1.028	0.324**	1.030	0.709***

*Note:* Results of six linear regression models with different dependent variables, but the same 11 independent variables. *B* and *β* are used to represent the unstandardised regression coefficients and standardised regression coefficients, respectively. Significance was tested two‐tailed.

Abbreviations: MSPSS, Multidimensional Scale of Perceived Social Support; PCS, Pain Catastrophizing Scale; PHQ‐9, Patient Health Questionnaire; POD, post‐operative day.

^a^ *p* values are based on bias‐corrected and accelerated bootstrap with 2000 samples.

^b^ Adjusted *R* ^2^ = 0.16, *F*_(11,186)_ = 4.36, *p* < 0.001.

^c^ Adjusted *R* ^2^ = 0.13, *F*_(11,183)_ = 3.71, *p* < 0.001.

^d^ Adjusted *R* ^2^ = 0.19, *F*_(11,186)_ = 5.16, *p* < 0.001.

^e^ Adjusted *R* ^2^ = 0.07, *F*_(11,183)_ = 2.36, *p* < 0.01.

^f^ Adjusted *R* ^2^ = 0.20, *F*_(11,185)_ = 5.32, *p* < 0.001.

^g^ Adjusted *R* ^2^ = 0.57, *F*_(11,183)_ = 24.50, *p* < 0.001.

* *p* < 0.05.

** *p* < 0.01.

*** *p* < 0.001.

To test if separate sources of support were better predictors than the total scale, we exploratorily ran additional regression models for pain at rest and pain at movement on POD7, respectively, with the MSPSS subscales. None of the subscales significantly predicted pain at rest or pain at movement on POD7 in these post hoc analyses.

## 4 Discussion

In this study, we described high levels of PSS in middle‐aged patients scheduled for surgery in a medium‐sized city in Germany (~130,000 inhabitants). PSS correlated significantly but weakly with pre‐operative chronic pain and opioid intake, symptoms of depression, pain‐catastrophising and socio‐demographic characteristics. Moreover, it did not predict acute post‐operative pain intensity and medication use. However, known risk factors for severe acute post‐operative pain could be confirmed.

### 4.1 High PSS in Our Cohort

PSS assessed by the MSPSS score turned out to be very good in our cohort. This must be viewed against the background that our data were collected in a relatively homogeneous society in a well‐off Bavarian university town. With an average score of 6.20 ± 1.02, PSS is considerably higher than in any study using the MSPSS known to us, with values ranging around 4.72 (Zimet et al. 1988), 5.58 (Dahlem et al. 1991) and 5.80 (Grey et al. 2020), to give some examples. All of these studies investigated a distinctly younger sample compared with our study, making our results particularly interesting, considering that previous studies found PSS to decrease with higher age (Prezza and Giuseppina Pacilli 2002). While age itself did not correlate significantly with the overall MSPSS score in our study, PSS from friends and significant others decreased with higher age. Considering the later discussed impact of marital status on PSS, we assume that personal living conditions influence PSS more strongly than age. High PSS values in our patients might be partially explained by this; however, a more important influential factor might be the timing of the survey just before a surgical intervention. For most patients, this is a very stressful experience and individuals with high levels of resilience can buffer the negative impact on mental and physical health more successfully (Cohen and Wills 1985; Flannergy and Wieman 1989). As social support is considered a key factor in building resilience, the high MSPSS scores could be explained in different ways: Relatives and friends could pay special attention to the patient before surgery, the patient could actively seek additional social support before surgery or the patient could simply be more aware of the available social support than they would be in everyday life. The high MSPSS scores suggest that a ceiling effect should be considered in the following interpretations.

### 4.2 Association of PSS With Various Patient Properties

We were able to show small but significant associations of PSS with numerous pre‐operatively assessed factors, including this study's strongest predictor of acute post‐operative pain, that is, pre‐operative chronic pain. While the negative correlation of PSS with chronic pain and associated psychological factors such as depression and anxiety had already been described in literature (Grey et al. 2020; Jensen et al. 2011; Shao et al. 2020), its remarkable correlation with marital status was surprising to us and could not previously be confirmed (Prezza and Giuseppina Pacilli 2002). This supports our assumption that the stage of life has a major influence on PSS, as marital status reflects one's current life situation more directly than age and appears to provide better access to PSS. Our data suggest that committed relationships are an important source of social support during a stressful experience, such as surgery.

### 4.3 Risk Factors for Severe Acute Post‐Operative Pain

We could confirm some of the predictors of more severe acute post‐operative pain described in the literature (Schnabel et al. 2020; Yang et al. 2019). Interestingly, the significant predictors for severe post‐operative pain and for high post‐operative opioid doses did not overlap. We believe that patients with chronic opioid use generally receive more intensive post‐operative analgesia than opioid‐naïve patients. We theorise as follows: On one hand, there are patients with pre‐operative chronic pain but without chronic opioid medication who are not recognised as chronic pain patients. Post‐operatively, these patients experience more pain but are not automatically treated with higher opioid doses. By POD7, pain medication has already been adjusted in response to the increased pain, which weakens the association between pre‐operative factors and post‐operative pain compared to POD1. On the other hand, patients who are on long‐term opioid therapy receive higher post‐operative doses by default and might be monitored more closely. This results in the pre‐operative morphine equivalent dose predicting the post‐operative morphine equivalent dose, but not the post‐operative pain intensity.

### 4.4 Independency of Acute Post‐Operative Pain From PSS

The small, but significant correlations between PSS and lower pain at rest on POD7 and less opioid intake on POD1, respectively, partially confirm our hypothesis of social support's beneficial effects. In multiple regression models, however, PSS did not significantly predict the severity of acute post‐operative pain or the amount of opioid administered on POD1 and POD7 beyond previously established risk factors. We consider this a sound conclusion, because a post hoc power calculation revealed that, even with multiple predictors, all our regression models had sufficient statistical power of < 0.9 (except 0.71 for the model predicting pain at movement on POD 7). Therefore, pre‐operative PSS assessed by the MSPSS is insufficient as part of risk scores for acute post‐operative pain intensity. Furthermore, our data provide only weak support for enhancing PSS as a therapeutic approach for the prevention of severe acute post‐operative pain.

The following considerations might be helpful to explain our findings in line with previous studies. PSS assessed before surgery might differ systematically from everyday‐life PSS, and the mechanisms by which general PSS modulates pain might differ from situation‐specific processes.

Previous research suggests that social support triggers feelings of safety, thereby reducing pain intensity (Reddan et al. 2020). People with high levels of general PSS might benefit from corresponding experiences and expectations when undergoing surgery. To effect acute post‐operative pain, however, situation‐specific, short‐term effects of immediate social support may be necessary. The fact that we assessed social support only on a general level, rather than situation specific, may explain why our results diverge from previous studies reporting lower pain intensities when partner showed affection or was simply present (Eisenberger 2012; Master et al. 2009; Reddan et al. 2020). Future studies should use more fine‐grained measures of social support, for example based on smartphone applications (Weiss, Gründahl, et al. 2024; Weiss, Jachnik, et al. 2024) that can assess perceived social support in daily life and clinical setting, and potentially capture pre‐to‐post operative changes in PSS. Moreover, future studies could investigate differences in pain intensity depending on the presence of a significant other to verify the applicability of the mentioned findings to real patients. Furthermore, they might confirm the previously reported positive effect of high PSS on acute pain unpleasantness in a more diverse cohort (Munk et al. 2023). The role of hospital staff as a source of social support could also be examined in this context. However, we estimate the importance of PSS to be higher for chronic post‐operative pain and recommend future researchers to investigate this further.

### 4.5 Limitations

The following limitations need to be considered. First, varying time intervals between the pre‐operative assessment and surgery could have biased the results. Second, we did not document all the patients who refused to participate in the study from the beginning. However, the 58 patients we did document showed no substantial difference in age and sex compared with our cohort. Third, we did not check for general mood nor for a possible social desirability bias when completing the MSPSS, but studies imply that these two factors can be ignored (Askim and Knardahl 2021; Dahlem et al. 1991). And finally, we performed a multiple regression analysis by including many risk factors beyond social support, which might have led to overfitting of these results in our sub‐cohort of patients.

## 5 Conclusion

As the first study to investigate the role of pre‐operatively assessed PSS on post‐operative acute pain intensity in a diverse patient population, we were able to show its association with known risk factors for severe post‐operative acute pain. However, PSS assessed by the MSPSS before surgery did not emerge as an additional predictor of post‐operative pain or the amount of post‐operatively administered opioids, which precludes its use as a risk factor and the modulation of PSS as a therapeutic approach. Future studies are needed to elucidate how PSS affects our perception of pain.

## Author Contributions

K.T., K.S., M.F., F.F., D.C.R., E.M.P.‐Z. and H.L.R. designed the study. P.W. collected and analysed the data and drafted the manuscript. K.T., M.G., G.H. and H.L.R. substantially contributed to the interpretation of the data. All authors revised the manuscript critically for important intellectual content and approved the submitted version.

## Consent

The authors have nothing to report.

## Conflicts of Interest

E.M.P.‐Z. received financial support from Gruenenthal for research activities and advisory and lecture fees from Gruenenthal, MSD/Merck and Medtronic. In addition, she received scientific support from the German Research Foundation (DFG), the Federal Ministry of Education and Research (BMBF), the Federal Joint Committee (G‐BA) and the Innovative Medicines Initiative 2 Joint Undertaking under grant agreement no. 777500. This Joint Undertaking received support from the European Union's Horizon 2020 research and innovation programme and EFPIA. All money went to the institutions E.M.P.‐Z. is working for. H.L.R. received funds from Gruenenthal and Orion for consultation unrelated to the study and research support by the German Research Foundation (DFG), the Federal Ministry of Education and Research (BMBF) and the Federal Joint Committee (G‐BA) independent of the study here. K.T., M.G., P.W., K.S., D.C.R., F.F. and M.F. declare that they have no competing interests.

## Data Availability

The datasets analysed during the current study are available from the corresponding author on reasonable request.
